# Prevalence of cardiometabolic risk factors in a nationally representative sample of Iranian children and adolescents: the CASPIAN-V Study

**DOI:** 10.15171/jcvtr.2018.12

**Published:** 2018-05-31

**Authors:** Mohammad Esmaeil Motlagh, Mostafa Qorbani, Amir-Masood Rafiemanzelat, Majzoubeh Taheri, Tahereh Aminaee, Gita Shafiee, Zeinab Ahadi, Mahshid Hajiali, Kimia Ghaderi, Ali Safaei, Azam Goodarzi, Hasan Ziaodini, Ramin Heshmat, Roya Kelishadi

**Affiliations:** ^1^Bureau of Family, Population, Youth and School Health, Ministry of Health and Medical Education, Tehran,Iran; ^2^Department of Pediatrics, Ahvaz Jundishapur University of Medical Sciences, Ahvaz, Iran; ^3^Non-communicable Diseases Research Center, Alborz University of Medical Sciences, Karaj, Iran; ^4^Student Research Committee, School of Medicine, Isfahan University of Medical Sciences, Isfahan, Iran; ^5^Office of Adolescents and School Health, Ministry of Health and Medical Education, Tehran, Iran; ^6^Chronic Diseases Research Center, Endocrinology and Metabolism Population Sciences Institute, Tehran University of Medical Sciences, Tehran, Iran; ^7^Department of Cardiology, Shahid Beheshti University of Medical Sciences, Tehran, Iran; ^8^Department of Health Education and Promotion, Tarbiat Modarres University, Tehran, Iran; ^9^Health Psychology Department, Research Center of Education Ministry Studies, Tehran, Iran; ^10^Pediatrics Department, Child Growth and Development Research Center, Research Institute for Primordial Prevention of Noncommunicable Disease, Isfahan University of Medical Sciences, Isfahan, Iran

**Keywords:** Cardiometabolic, Prevalence, Children, Adolescents

## Abstract

***Introduction:*** This study presents the prevalence of cardiometabolic risk factors in a nationally representative sample of Iranian children and adolescents.

***Methods:*** This multi-centric study was conducted in 2015 among 4200 students aged 7–18 years. They were selected by multistage cluster sampling from 30 provinces of Iran. Anthropometric indices, biochemical and clinical parameters were measured.

***Results:*** The mean of weight, height, waist circumference (WC), systolic blood pressure (SBP), diastolic blood pressure (DBP), and fasting blood glucose (FBG) was higher in boys than in girls (*P * < 0.05). The mean of triglyceride (TG), total cholesterol (TC) and low-density lipoprotein (LDL) levels were higher in girls than in boys (*P * < 0.05). The mean of weight, height, WC, SBP, DBP, alanine transaminase (ALT) and body mass index (BMI) was higher in urban than in rural residents (*P * < 0.05). Overall, 16.1%, 9.4% and 11.4% were underweight, overweight and obese. Abdominal obesity was documented in 21.6% of boys and 20.5% of girls. Low HDL-C was the most prevalent abnormality of lipid profile (29.5%) followed by high serum TGs (27.7%). Low HDL-C was more prevalent in boys than in girls (32.7% vs. 26%, respectively, *P * < 0.05). Prevalence of obesity and overweight were higher in girls than in boys (*P * < 0.05). The prevalence of obesity and overweight, abdominal obesity, and low HDL-C were higher in urban than in rural residents (*P* < 0.05).

***Conclusion:*** We found considerably high prevalence of some cardiometabolic risk factors including overweight and obesity, low HDL-C and hypertriglyceridemia in Iranian children and adolescents. The current findings underscore the necessity of intensifying health interventions for primordial and primary prevention of non-communicable diseases from early life.

## Introduction

Developing countries are struggling with health transition. Although in many of these communities, infectious diseases are still playing important role, rapid changes in lifestyle caused by high velocity of urbanization and globalization, may have led to non-communicable diseases (NCDs), as the most important cause of mortality.^1-3^

Among NCDs, cardiovascular diseases (CVD) are one of the most considerable causes of morbidity and mortality worldwide; being responsible of 50% of death at global level.^4^ It is well documented that atherosclerosis begins from early life and progresses over time.^5,6^ NCDs have common risk factors; thus screening and management of their modifiable risk factors is important in primordial/primary prevention programs.^7^ Several studies have indicated early life environmental factors as main factor in progression of chronic adulthood diseases.^5,8^ however the role of genetic factors should also be considered.^9^ Global studies have documented high concentration of low density lipoprotein- cholesterol (LDL-C), low concentration of high density lipoprotein- cholesterol (HDL-C), elevated blood pressure, elevated fasting blood glucose (FBG), and obesity as the most important cardiometabolic factors.^10,11^

Some studies have presented the effects of ethnic, racial and regional factors on the frequency of cardiometabolic risk factors. It is reported that total cholesterol had higher progression in high-income communities, and FBG had a more steeply rise in the Middle East and United States.^12^ Similar to many other Middle Eastern countries,^13^ the prevalence of cardiometabolic factors is considerably high in Iran.^14,15^

A nationwide school-based surveillance program, entitled Child and Adolescent Surveillance and Prevention of Adult Non-communicable diseases (CASPIAN), showed high prevalence of some cardiometabolic risk factors in Iranian pediatric population. It revealed that low level of HDL-C, hypertriglyceridemia and overweight were the most prevalent cardiometabolic factors in Iranian pediatric population.^16^

Given the rapid changes in environmental and lifestyle factors, this frequency may change over time. The current study presents the prevalence of cardiometabolic factors in the fifth phase of this surveillance program, conducted in a nationally-representative sample of Iranian children and adolescents.

## Materials and Methods

### Study Population

The data of the present study are as a part of Childhood and Adolescence Surveillance and Prevention of Adult Non-communicable Disease (CASPIAN-V) study. This study was conducted in 30 provinces in Iran that is school-based nationwide health survey. Protocol details of CASPIAN-V study have been explained before.^17^ The study population consisted of students aged 7-18 years living in urban and rural areas. Multistage stratified cluster sampling method was used for selecting subjects. Sampling within each province was conducted according to the student’s place of residence (urban or rural) and level of education (primary and secondary). The sample size of main survey included 14 400 students at national level. In each province, 14 out of 48 clusters were randomly selected for biochemical tests. Therefore, sample size of subjects with biochemical tests was estimated to be 4200.

The sample size for biochemical tests in this nationwide study was calculated as the maximum sample size needed to provide an optimal estimate of all the biochemical testes. The sample size was determined based on a proportion estimation formula. To obtain maximum sample size, prevalence of metabolic syndrome in the previous studies in Iranian children and adolescents^18,19^ was considered as 2.8%, precision as 0.5%, and type I error as 0.05. Using the multistage cluster random sampling, the students were selected from urban and rural areas in the 30 provinces (14 clusters of 10 students in each province). Stratification was done in each province according to the living area (urban/rural) and school grade (primary/secondary). The sampling was proportional to the size, with an equal sex ratio (the number of boys and girls was the same in each province). The clusters were concluded the level of schools and 10 sample units in each cluster was selected. Finally, 14 clusters of 10 students in each of the provinces (n: 4200 students) were selected.

### Anthropometric assessment

Weight was measured on a scale placed on a flat ground and light cloth with standard scale to the nearest 0.1 kg. Height was measured without shoes to the nearest 0.1 cm. Body mass index (BMI) was calculated by dividing weight (kg) to height squared (m^2^). We used the WHO growth charts to categorize BMI.^20^ Waist circumference (WC) was measured using a non-elastic tape at a point midway between the lower border of the rib cage and the iliac crest at the end of normal expiration to the nearest 0.1 cm.

### Biochemical assessment

To assess blood lipid levels, students were referred to the laboratory. Fasting venous blood samples was taken after 12 hours overnight fasting. All samples were stored at -70°C and finally were transferred by cold chain to Isfahan Mahdieh Laboratory. FBG, triglycerides (TG), total cholesterol (TC), low- density lipoprotein-cholesterol (LDL-C) and HDL-C were measured enzymatically by Hitachi auto-analyzer (Tokyo, Japan).^21^

### Statistical analysis

Data was analyzed by using STATA package version 11.0 (Stata Statistical Software: Release 11. College Station, TX: Stata Corp LP. Package), and *P* < 0.05 was considered as statistically significant. Continuous variables are expressed as mean and standard deviation (SD), and categorical variables as number (percentage). The Student t-test and analysis of variance (ANOVA) were used to compare mean differences between quantitative variables. Differences between qualitative variables were assessed by Pearson χ2 test.

## Results

Among 4200 selected students, participation rate of blood sampling was 91.5%. Table 1 presents the characteristics of study participants by sex, age and residence area. The mean (SD) of weight, height, WC, systolic blood pressure (SBP), DBP, and FBG was higher in boys than in girls (*P *< 0.05). The mean (SD) of TG, total cholesterol and LDL-C levels were higher in girls than in boys (*P *< 0.05). The mean (SD) of weight, height, WC, SBP, DBP and BMI was higher in urban than in rural residents (*P *< 0.05). The mean (SD) of weight, height, WC, SBP, DBP, and BMI increased significantly with rising age (*P *< 0.05).

**Table 1:** Characteristics of participants by age, gender, and living area: the CASPIAN-V Study

		**Weight**	**Height**	**WC**	**SBP**	**DBP**	**FBG**	**TG**	**Cholesterol**	**HDL**	**LDL**	**ALT**	**BMI**
		**Mean ± SD**											
Gender	Boy	42.36±18.23	148.15±18.77	67.65±12.87	99.55±13.43	64.08±10.70	92.06±12.91	87.15±45.52	152.96±28.06	46.21±10.17	89.31±22.90	8.49±5.11	18.48±4.96
	Girl	40.41±15.42	144.93±15.93	65.76±11.33	98.77±12.72	63.57±10.14	91.20±11.14	89.02±44.78	154.83±26.67	46.16±9.75	90.86±22.26	8.16±8.73	18.53±4.43
*P* value^*^		<0.001	<0.001	<0.001	<0.001	0.004	0.027	0.021	0.035	0.86	0.03	0.13	0.56
Residence area	Urban	43.66±17.73	148.62±17.74	68.27±12.27	100.15±13.05	64.28±10.45	91.76±12.56	88.47±45.54	153.77±27.64	46.25±10.19	89.83±22.81	8.41±7.90	18.98±4.83
	Rural	35.75±13.96	141.42±15.75	62.85±11.02	96.70±12.86	62.72±10.29	91.36±10.84	86.92±44.21	154.04±26.84	46.02±9.38	90.63±22.06	8.12±4.16	17.32±4.16
*P* value^*^		<0.001	<0.001	<0.001	<0.001	<0.001	0.35	0.33	0.78	0.51	0.32	0.13	<0.001
Age (y)	7-10	27.69±8.80	130.05±10.15	59.55±9.05	93.99±12.72	60.82±10.35	91.67±14.00	87.12±45.64	154.72±29.21	47.08±10.60	90.21±24.12	8.43±6.76	16.18±4.03
	11-14	42.05±13.11	148.85±11.79	67.47±11.18	99.43±12.33	63.94±9.97	91.77±11.33	87.76±44.37	154.07±26.30	46.04±9.75	90.47±21.69	8.26±4.79	18.66±4.41
	15-18	57.68±15.43	164.01±12.27	74.66±11.70	105.25±11.89	67.44±10.01	91.44±11.02	89.50±45.94	152.53±27.10	45.43±9.54	89.20±22.31	8.34±9.88	21.21±4.42
*P* value^**^		<0.001	<0.001	<0.001	<0.001	<0.001	0.79	0.44	0.16	<0.001	0.34	0.83	<0.001
Total		41.39±17.11	146.56±17.50	66.72±12.17	99.17±13.09	63.83±10.43	91.65±12.11	88.04±45.18	153.85±27.42	46.19±9.97	90.05±22.60	8.33±7.07	18.51±4.71

Abbreviations: WC, waist circumference; SBP, systolic blood pressure; DBP, diastolic blood pressure; FBG, fasting blood glucose; TG, triglyceride; ALT, alanine transaminase; BMI, body mass index; HDL, High-density lipoprotein; LDL, Low-density lipoprotein. ^*^According to independent sample t-test ^**^According to ANOVA test *P*< 0.05 is significant.

Table 2 shows the prevalence of different cardiometabolic risk factors according to gender, living area and age group. Low HDL-C was more prevalent in boys than in girls (32.7% vs. 26%, respectively, *P *< 0.05). Other variables were not significantly different in terms of gender. Prevalence of obesity and overweight were higher in girls than in boys (*P *< 0.05). The prevalence of obesity and overweight, abdominal obesity, and low HDL-C were higher in urban than in rural residents (*P *< 0.05).

**Table 2:** Prevalence of cardiometabolic risk factors by age, gender, and living area: the CASPIAN-V study

		**Total**	**Gender, No. (%)**			**Residence area, No. (%)**			**Age, No. (%)**			
			**Boys**	**Girls**	***P*** ** value** ^*^	**Urban**	**Rural**	***P*** ** value** ^*^	**7-10 y**	**11-14 y**	**15-18 y**	***P *** **value** ^*^
Weight status	Underweight	2279 (16.1)	1249 (17.4)	1030 (14.8)	<0.001	1443 (14.3)	810(20)	<0.001	900(18.8)	861(15.5)	492(12.9)	<0.001
	Normal	8914 (63)	4403 (61.4)	4511 (64.7)		7675 (76)	3030 (74.9)		3455(72.1)	4192(75.6)	3058(80.3)	
	Overweight	1330 (9.4)	621 (8.7)	709 (10.2)		266 (2.6)	53 (1.3)		131(2.7)	119(2.1)	69(1.8)	
	Obese	1615 (11.4)	896 (12.5)	719 (10.3)		711 (7)	153 (3.8)		305(6.4)	372(6.7)	187(4.9)	
Abdominal obesity		2972 (21.1)	1550 (21.6)	1422 (20.5)	0.08	2335 (23.2)	637 (15.8)	<0.001	974(20.4)	1156(20.9)	842(22.2)	0.11
High TG		1065 (27.7)	541(26.9)	524 (28.6)	0.22	782 (28.2)	283 (26.5)	0.30	299(26.1)	457(27.6)	309(29.7)	0.17
High LDL		674 (17.5)	341(16.9)	333 (18.2)	0.31	489 (17.6)	185 (17.3)	0.83	208(18.1)	282(17)	184(17.7)	0.74
High TC		189 (4.9)	100 (5)	89 (4.9)	0.88	140(5)	49 (4.6)	0.56	72(6.3)	74(4.5)	43(4.1)	0.04
Low HDL		1134 (29.5)	658 (32.7)	476 (26)	<0.001	849 (30.6)	285 (26.7)	0.02	275(24)	428(25.9)	431(41.4)	<0.001
High SBP		438 (3.1)	210 (3)	228 (3.3)	0.25	330 (3.3)	108 (2.7)	0.07	229(4.8)	158(2.9)	51(1.4)	<0.001
High DBP		1450 (10.4)	746 (10.5)	704 (10.2)	0.51	1053 (10.5)	397 (10)	0.32	427(9)	729(13.2)	294(7.8)	<0.001
High blood pressure		1604 (11.5)	815 (11.5)	789 (11.4)	0.87	1169 (11.7)	435 (10.9)	0.19	515(10.9)	775(14.1)	314(8.3)	<0.001
High FBG		161 (4.2)	96 (4.8)	65 (3.5)	0.06	116 (4.2)	45 (4.2)	0.96	47 (4.1)	71 (4.3)	43 (4.1)	0.96

Abbreviations: SBP, systolic blood pressure; DBP, diastolic blood pressure; FBG, fasting blood glucose; TG, triglyceride; HDL, High-density lipoprotein; LDL, Low-density lipoprotein; TC, total cholesterol. ^*^According χ^2^ test.

The prevalence of elevated SBP was higher in those aged 7-10 years, whereas elevated BP and DBP were more prevalent in subjects with 11-14 years of age, and the prevalence of low HDL-C was higher in subjects aged 15-18 years than in other age groups (*P *< 0.05).

## Discussion

This multicentric study investigated the prevalence of cardiometabolic factors, conducted in a nationally-representative sample of Iranian children and adolescents.

A study on developing countries has reported the global prevalence of excess weight, i.e. obesity plus overweight is 10%,^22^ ranging from 5.7% in Pakistan^23^ to 40% in Mexico.^24^ Our findings indicate 9.1% (2.7% overweight and 6.4% obese) among 7-10 years children, 8.8% (2.1% overweight and 6.7% obese) among 11-14 and 6.7% (1.8% overweight and 4.9% obese) among 15-18. The prevalence of obesity in Iran is less than globe, as compared with above. In the present study, the prevalence of obesity and overweight was higher among girls that is in accordance with the study on school children and adolescents in Chennai^25^ but in contrast with the study on school children and adolescents in south Mumbai.^26^

The number of studies in which BMI status has been compared between urban vs. rural residents is low. However, studies that were conducted in different areas of India, demonstrated children living in urban areas had significantly higher rate of both overweight and obesity^27,28^ which is in accordance with our study. Higher prevalence of obesity among urban children may correlate with lower quality of life style and dietary patterns in urban areas. A previous study demonstrated cardiometabolic risk factors were different according gender, living area and age group.^16^

As WC has been proved to be an independent risk factor of NCDs in adults, it has been assessed in our study as well. In urban areas, 23.2% of children had high WC while this rate was 15.8% in rural residents.

Lipid profiles are other factors explained for metabolic syndrome.^5^ Moreover, the association between childhood obesity and lipid disorders is well documented.^29^ Consistent with some previous studies in Iran, low HDL was the most prevalent dyslipidemia associated risk factor of metabolic syndrome, followed by high triglyceride and high LDL.^16^ A study has reported slight decline in total cholesterol of children form 1980-2008, while dyslipidemia has been increasing in east and south east of Asia.^30^ A study in China reported 16.1% of high triglyceride, 6.4% of low HDL and 5.8 % of high LDL among 6-18 years children.^31^ However, this finding is in contrast with high income nations such as Japan and United States that have not reported such increase in lipid profiles.^32,33^ High level of LDL has increased in comparison to previous conducted study in Iranian children and adolescents.^16^ Study on Brazilian school children showed dyslipidemia was associated with the geographic location of school and WC of child. In addition, high blood glucose was correlated with the school’s geographic location, peripheral adiposity of child and age.^34^ According to one cross-sectional study, children below ten years old, with higher-income families and with higher maternal schooling had greater risk for developing overweight, hypercholesterolemia and hypertriglyceridemia.^35^

Another prognostic factor of CVDs is blood pressure. An estimated prevalence of worldwide high systemic blood pressure among children is 1%-13%.^36^ A study among Portuguese children reported similar prevalence rate, ranging from 11.2% in normal-weight children to 39.7% in obese children.^37^ A study on Lithuanian children reported 25.1% of systemic hypertension among 12–15 year-old. These finding showed boys were more affected with high blood pressure than girls and also it was more prevalent among older children.^38^ Our findings are not consistent with these studies; although mean SBP and DBP is lower in the current study compared with a previous study in Iran^16^ the rate of high blood pressure with regards to different variables including age, sex and residence had significant increase.

There are some limitations in the present study. First, the cross-sectional design of the CASPIAN study. Second, CVD is multifactorial and other factors such as family history and behaviors including diet, physical activity, and smoking are also important. Advantages of the study are its large sample size and assessment of risk factors according to age, gender, and living area.

## Conclusion

We found considerably high prevalence of some cardiometabolic risk factors including overweight and obesity, low HDL-C and hypertriglyceridemia in Iranian children and adolescents. The current findings underscore the necessity of intensifying health interventions for primordial and primary prevention of NCDs from early life.

## Ethical approval

This study was approved by ethical committees and other relevant national regulatory organizations. The Research and Ethics council of Isfahan University of Medical Sciences approved the study (Project number: 194049). Written informed consent and verbal consent were obtained from the parents and students, respectively.

## Competing interests

None.

## Acknowledgements

The authors are indebted to all the participants for their dedicated and conscientious collaboration. This study was not supported by any fund.
